# Clinical review is essential to evaluate 30-day mortality after trauma

**DOI:** 10.1186/1757-7241-22-18

**Published:** 2014-03-13

**Authors:** Poya Ghorbani, Magnus Falkén, Louis Riddez, Martin Sundelöf, Anders Oldner, Lovisa Strömmer

**Affiliations:** 1 Division of Surgery, Department of Clinical Science, Intervention and Technology (CLINTEC), Karolinska Institute, Stockholm, Sweden; 2 Department of Molecular Medicine and Surgery, Karolinska Institute, Stockholm, Sweden; 3 Division of Anesthesiology and Intensive care, Department of Physiology and Pharmacology, Karolinska Institute, Stockholm, Sweden; 4 P9:03, Department of Surgical Gastroenterology, Karolinska University Hospital, Solna 171 76 Stockholm, Sweden

**Keywords:** Mortality, Trauma deaths, Clinical review, Dead on arrival

## Abstract

**Background:**

Securing high-quality mortality statistics requires systematic evaluation of all trauma deaths. We examined the proportion of trauma patients dying within 30 days from causes not related to the injury and the impact of exclusion of patients dead on arrival on 30-day trauma mortality. We also defined the demographics, injury characteristics, cause of death and time to death in patients admitted to our trauma center who died within 30 days, between 2007-2011.

**Methods:**

Demographics, injury characteristics, status alive/dead on arrival, cause of death and time to death of all patients were reviewed. Deaths were analyzed based on injury mechanism (penetrating, blunt trauma and low energy blunt trauma) and cause of death (traumatic brain injury (TBI), hemorrhage, organ dysfunction and other/unknown).

**Results:**

Of the 7422 admissions, 343 deaths were identified of which 36 (10.5%) involved causes not related to the injury. The overall age was 71 years, Injury Severity Score (ISS) 29 and time to death 24 hours (all medians). Fifty-four patients (17.6%) were dead on arrival. Exclusion of patients dead on arrival reduced the overall mortality rate (P < 0.05) and median ISS (P < 0.05) and increased median age (P < 0.01) and time to death (P < 0.001). Injury mechanism was penetrating trauma in 7.5%, blunt trauma in 56.0%, and low energy blunt trauma in 36.5%. TBI accounted for 58.6%; hemorrhage 16.3%, organ dysfunction 15.0%, and other/unknown for 10.1% of the deaths. Patients who died after low energy blunt trauma were older, had lower ISS and longer time to death compared to those who died after penetrating and blunt trauma (all P < 0.01).

**Conclusions:**

Clinical review of all trauma deaths was essential to interpret mortality. Thirty-day trauma mortality included 10.5% deaths not directly related to the injury and the exclusion of patients dead on arrival significantly affected the unadjusted mortality rate, ISS, median age and time to death.

## Background

Mortality is used as an outcome parameter of trauma care. High quality statistics on mortality is essential for improving the quality of care and requires systematic evaluation of all trauma deaths [1-3]. However, variations in the definitions and methodologies used may have consequences for the interpretation of trauma mortality.

Thirty-day mortality is a recommended endpoint in trauma research [4,5], but deaths at 30 days after trauma may include patients dying from causes not directly related to the previous injury. Variations in the definition and exclusion of patients dead on arrival (DOA) may also distort interpretation of mortality outcomes [6,7]. Explicit criteria to declare a patient DOA have been suggested [8], but no definition of DOA has been universally adopted in trauma research. To use only autopsy data [9] or death certificates [10] may be a source of bias in injury research, but abstracting data from several sources in combination with individual review increases the likelihood of determining the appropriate cause of death.

In the current study, we examined the proportion of trauma patients dying within 30 days from causes not directly related to the injury and the impact of exclusion of patients dead on arrival on 30-day trauma mortality. We also defined the demographics, injury characteristics, cause of death and time to death in patients admitted to our trauma center who died within 30 days, during a five year period.

## Methods

### Setting

Prospectively collected data from the 2007–2011 trauma registry at the Karolinska University Hospital, Stockholm, Sweden were analyzed retrospectively. Since 2007, this hospital has served as the only primary trauma care facility for two million inhabitants. The pre-hospital care system is based on paramedic and nurse-manned ambulances, anesthesiologist-manned rapid response care and a helicopter system. Trauma trained surgeons and consultant specialists are located within the hospital.

### Data collection and analysis

All patients ≥15 years who died within 30 days were identified from the trauma registry. The local trauma registry records all patients admitted with trauma team activation and patients without trauma team activation who have an ISS of >9. Data entry in the register has been described previously [11]. Demographic information, injury type and mechanism, ISS (AIS 05), comorbidity (ASA class) were collected and medical records reviewed. SOFA (Sequential-related Organ Failure Assessment) scores [12] were collected for deaths in the intensive care unit. The underlying cause of death according to the codes in International Classification of Diseases, tenth version (ICD-10) and the basis for the cause of death were collected from the Cause of Death Registry (CDR) at the National Board of Health and Welfare, Stockholm, Sweden.

### Trauma deaths and deaths not directly related to the injury

The review process was performed by two independent surgeons; disagreements were discussed with a third party and consensus reached.

The cause of death was based on the ICD-10 code and the clinical review. A trauma death was defined as an ICD-10 trauma code based on an autopsy; or an ICD-10 trauma code without an autopsy in which the clinical review supported a trauma death; or an ICD-10 non-trauma code in which clinical review supported a trauma death or could not rule out that trauma contributed to death. A death not directly related to the injury was defined as an ICD-10 non-trauma code based on an autopsy; or an ICD-10 non-trauma code without an autopsy in which the clinical review supported a death not directly related to the injury. If clinical review was not possible because of missing medical records, it was classified as a trauma related death in the Other/Unknown category if the ICD-10 code was trauma code. If the ICD-10 code was non-trauma code, the death was classified as a death not related to the injury.

### Dead on arrival

Patients declared DOA were defined both by clinical review and by the explicit criteria for DOA [8]: blunt trauma patient arriving with no signs of life (pupillary response, respiratory effort, or motor activity) and prehospital CPR >5 min; or penetrating trauma with no signs of life, asystole without the possibility of cardiac tamponade and prehospital CPR >15 min.

### Definition of the cause of death

Traumatic brain injury (TBI) was defined as a cerebral, brainstem or high spinal injury incompatible with life. Hemorrhage was clinically documented and led to a complete loss of blood volume or hypovolemic arrest. Death was attributed to organ dysfunction (OD) when clinical documentation or the SOFA score supported organ failure (alone or in combination). Other/Unknown (O/U) deaths were those where there were other causes of death, or where the cause of death could not be established.

### Ethical approval

This study was approved by the Regional Ethical Review Board in Stockholm, Sweden.

### Statistics

Data are presented as median and interquartile range. The Mann–Whitney test was used for continuous data; Fisher’s exact test or chi-square test was used for categorical data. For comparisons of continuous data between more than two groups, analysis of variance was followed by Dunn’s test for multiple comparisons. Statistical significance was defined as P < 0.05.

## Results

There were 7422 trauma admissions, 1626 involving patients with ISS >15. A total of 343 patients were registered as dead during the study period, of whom 285 had ISS >15. Overall unadjusted mortality was 4.6% (343/7422) and mortality for ISS >15 was 17.5% (285/1626).

Eight deaths occurred outside the hospital. Only in two deaths were the medical records unavailable and clinical review was not possible. Of the 343 patients, 334 were identified in the CDR. The other nine patients were non-Swedish citizens and clinical review confirmed trauma deaths. Of the 334 patients identified in the CDR, 266 patients had ICD-10 trauma codes and 68 patients had non-trauma ICD-10 codes. Clinical review of these patients revealed that 265 of the 266 with trauma codes and 33 of the 68 without trauma codes had died of trauma-related causes. Conversely, 1 of 266 and 35 of 68 had died of causes not related to the injury. The final number of trauma deaths and deaths not related to the injury was 307 and 36 respectively.

### Deaths not related to the injury

Thirty-six trauma deaths (10.5%, 36/343) were not related to the injury (non-traumatic intracerebral bleeding n = 16, stroke n = 3, ischemic heart disease or myocardial infarction n = 6, cancer death n = 4, intoxication n = 2, pneumonia n = 1, bleeding stomach ulcer n = 1, terminal kidney disease n = 1, convulsions and asystole of unknown cause n = 2). This group consisted of older patients with a median age of 78 (62–88) years. Median ISS was 2 (1–6) representing minor injuries such as scalp wounds, hematomas or skin abrasions. The majority of the deaths occurred in hospital. Only two patients died outside the hospital after recovering from their injuries.

### All trauma deaths

The demographics of all trauma deaths (n = 307) excluding deaths not related to injury but including patients DOA is presented. The male:female ratio was 218:89. The overall median age was 64 (38–81) years. The median ISS was 29 (25–50) and time to death was 24 hours (3 hours-6 days).

### Injury mechanisms

**Figure 1:**
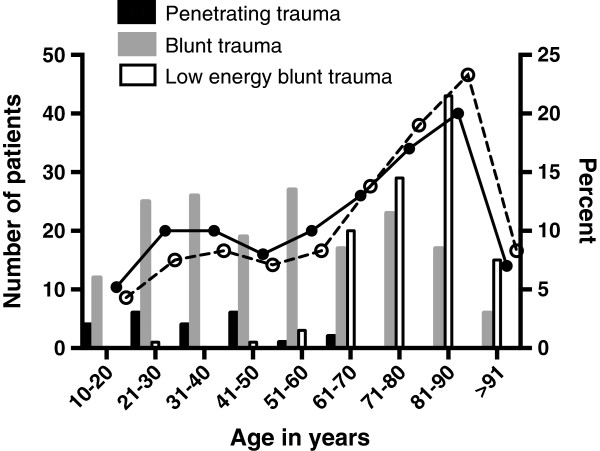
Number of deaths from penetrating (n = 23), blunt (n = 172) and low energy blunt trauma (n = 112) and percentage of all deaths (• symbol and solid line) and all deaths excluding patients DOA (ο symbol and dotted line) versus age group.

Deaths after penetrating trauma were due to stabbings (12/23), gunshots (10/23) and other penetrating object (1/23). In deaths after blunt trauma, motor vehicle accidents accounted for 32.4% (92/284), falls for 18.7% (53/284), falls from the same height for 29.2% (83/284), falls in stairs for 10.2% (29/284), other causes for 5.3% (15/284) and unknown for 4.2% (12/284). Deaths due to low energy blunt trauma (LEBT), i.e. falls from same height or falls in stairs, accounted for 39.4% of all blunt trauma deaths (112/284). The other deaths after blunt injury were called blunt trauma (BT, 172/284). Age, sex distribution and ISS for victims of LEBT, BT and penetrating trauma are shown in Table 1. The age distribution per injury mechanism is shown in Figure 1. Among the LEBT deaths, 87.5% (98/112) of the patients were ≥65 years and the major cause of death was TBI (92.8%, 104/112). ASA class 3 or 4 was more common in the LEBT group (58.6%, 65/112) compared to the BT group (19.5%, 26/132) and the ASA class median among the LEBT deaths [3 (2–3)] was higher compared to the median of 1 (1–2) in the BT deaths (both P < 0.0001).

**Table 1:** Patient characteristics subdivided by injury mechanism

	**LEBT**	**BT**	**PEN**
Deaths, n	112	172	23
Autopsy	16 (14.3%)	137 (79.7%)	23 (100%)
DOA	0	40	14
Age, years	81 (78–87)^a^	52 (33–73)^b^	33 (22–44)
Sex, male %	57%	77%	91%
ISS	25 (19–26)^a^	41 (27–59)	33 (26–51)
Time to death, hours	72 (20–192)^a^	13 (0.8–96)^b^	0.8 (0.3–4)

Values are median (interquartile range) unless otherwise indicated. LEBT–low energy blunt trauma, BT–blunt trauma, PEN–penetrating trauma. ISS = Injury Severity Score. DOA = Dead On Arrival.

^a^P < 0.001 vs BT and PEN, ^b^P < 0.01 vs PEN.

### Causes of deaths

**Figure 2:**
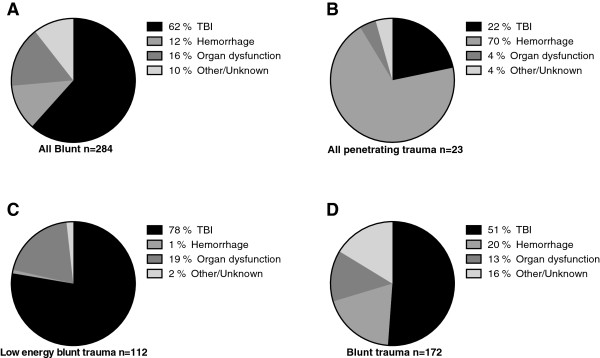
Causes of death in deaths owing to blunt trauma (A), penetrating trauma (B), low energy blunt trauma (C) and other blunt trauma (D).

TBI accounted for 58.6%; hemorrhage 16.3%, OD 15.0%, and O/U for 10.1% of all 307 deaths. TBI as the cause of death was more common for blunt than for penetrating trauma (62% vs 22%, P < 0.01, Figure 2), and hemorrhage was more common for penetrating than for blunt trauma (70% vs 12%) (P < 0.0001). There were differences in death cause frequencies between the BT and LEBT group, for TBI (51 vs 78%), hemorrhage (19 vs 1%) and O/U deaths (16 vs 2%) (all P < 0.0001). The median age of patients who died due to TBI and OD was higher and ISS lower (both P < 0.01 and P < 0.0001, respectively) than that of patients whose deaths were due to hemorrhage (Table 2). Among deaths ≥65 years, the cause of death was TBI in 76.3% (n = 75) and organ dysfunction in 20.4% (n = 20). In the OD group, 65% were initially cared for in the intensive care unit, but a majority (61%, 28/46) died in a surgical ward. Sixteen patients (35%, 16/46) fulfilled the SOFA criteria of multiple organ failure (MOF) and four of these patients were subjected to extracorporeal membrane oxygen treatment due to acute respiratory and/or cardiovascular failure. The majority of the deaths in the OD group (56%, 26/46) were due to cervical spine fractures and/or multiple rib fractures followed by respiratory and/or circulatory insufficiency with an early limitation of medical therapy in all patients. The final four patients in the OD group died due to asystole from unknown cause; they were not resuscitated. The O/U group of patients consisted of 31 patients of whom 15 patients were DOA. In the 16 patients not DOA, the cause of death was hanging and subsequent asphyxia in five patients. Among the other eleven patients, six deaths occurred outside the hospital. In nine patients, the cause of death could not be determined by clinical review and in two patients clinical review was not possible due to missing medical records.

**Table 2:** Patient characteristics subdivided by cause of death

	**TBI**	**Hemorrhage**	**Organ dysfunction**	**Other/Unknown**
Deaths, n	180	50	46	31
Autopsy	86 (47.8%)	50 (100%)	11 (23.9%)	29 (93.5%)
Penetrating: Blunt trauma	5:175	16:34	1:45	1:30
DOA	17 (9.4%)	22 (44.0%)	0 (0%)	15 (48.4%)
Age, years	69 (50–81)^a^	33 (24–50)	83 (70–88)^b^	53 (38–75)
Sex, male	65%	82%	72%	87%
ISS	27 (25–41)^a^	56 (39–75)	25 (17–30)^b^	38 (29–66)
Time to death, hours	28 (7–120)^c^	1 (0.3–3)	180 (72–312)^b^	0.8 (0.3–96)

Values are median (interquartile range) unless otherwise indicated. ISS = Injury Severity Score. DOA = Dead On Arrival.

^a^P < 0.01 vs hemorrhage, ^b^P < 0.0001 vs hemorrhage and Other/Unknown.

^c^P vs hemorrhage, organ dysfunction and Other/Unknown.

### Temporal distribution of death

**Figure 3:**
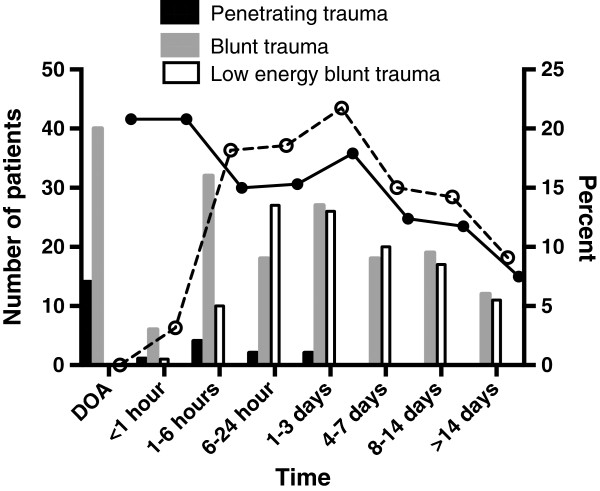
Number of deaths from penetrating (n = 23), blunt (n = 172) and low energy blunt trauma (n = 112) and percentage of all deaths (• symbol and solid line) and all deaths excluding patients DOA (ο symbol and dotted line) versus time intervals.

**Figure 4:**
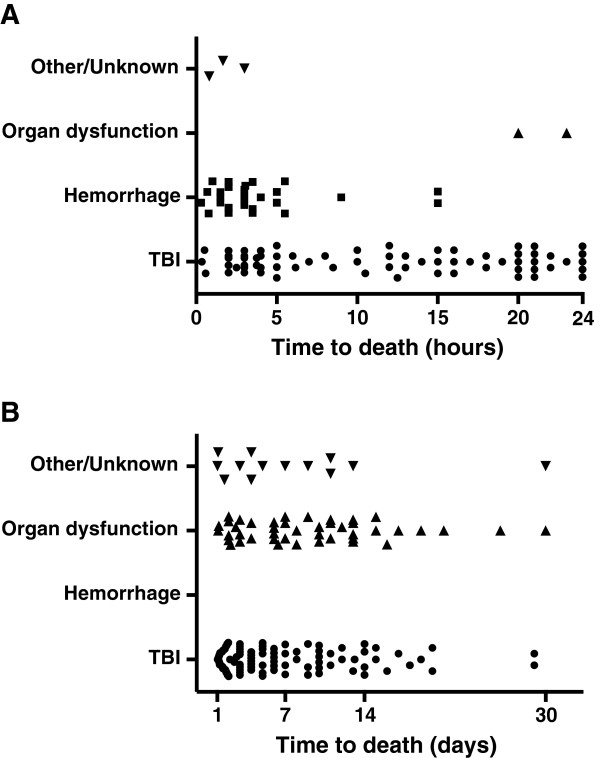
Time of deaths ≤24 hours (n = 101) (A) and >24 hours to 30 days (n = 152) (B) of patients dying from TBI, Hemorrhage, Organ dysfunction or Other/Unknown with patients DOA (n = 54) excluded.

The median time to death, in groups subdivided by injury mechanism and cause of death, is shown in Tables 1 and 2. Figure 3 shows time to death in time-intervals described by others [13]. Deaths ≤24 hours after admission accounted for 39.9% (101/253) of all deaths and the most common cause of death was TBI in 67.3% (68/101) followed by hemorrhage in 27.7% (28/101) (Figure 4). Two patients in the OD group died ≤24 hours after admission; both these patients were 90 years old and died of cardiac arrest and respiratory insufficiency, respectively. Of the patients who died from hemorrhage, 94.0% (47/50) died in less than six hours and 100% within 24 hours of admission. Deaths due to TBI occurred during the whole 30-day period with 91.4% (149/163) within 14 days (Figure 4). Deaths in the OD and O/U groups occurred within 14 days in 82.6% (38/46) and 93.8% (15/16) of the patients, respectively.

### Patients DOA

Clinical review classified 17.6% of the patients (54/307) as DOA. Forty-nine of these 54 patients (90.7%) fulfilled the explicit criteria set for DOA by other researchers [8], the other five patients failed to fulfill the criteria of DOA because of the absence of pre-hospital CPR.

Autopsy was performed on all patients. Causes of death were TBI (n = 17), hemorrhage (n = 22) and Other/Unknown (n = 15). Median age in the three cause of death groups TBI, hemorrhage and O/U was 44 (27–64), 33 (24–46) and 54 (32–69) years, respectively. Median ISS for TBI, hemorrhage and other/unknown causes of death was 51 (41–71), 50 (33–75) and 66 (41–75) years, respectively. There were no differences in median age or ISS between these three cause of death groups of patients DOA. The median time to the patient being declared dead from admission was 17 (10–26) minutes.

### The impact of patients DOA on mortality rate, age, distribution of causes of death and time to death

Unadjusted mortality was 4.2% including (307/7422) and 3.4% (253/7368) excluding DOA patients (P < 0.05). Two patients DOA had ISS <15. Mortality in patients with ISS >15 was 17.5% (285/1626) and showed a trend to a reduction to 14.8% (233/1574) when patients DOA were excluded (P = 0.084). After exclusion of patients DOA, the median age increased from 64 (38–81) to 71 (47–84) years (P < 0.01) and the median ISS decreased from 29 (25–50) to 27 (25–41) (P < 0.05). Exclusion of DOA did not significantly alter the cause of death (TBI 58.6% to 64.4%; hemorrhage 16.3% to 11.1%, OD 15.0% to 18.2% and O/U 10.1% to 6.3%). The median time to death in all trauma deaths almost doubled from 24 hours (3 hours-6 days) to 43 hours (12 hours-7 days) (P < 0.001) when patients DOA were excluded. If patients DOA were excluded from the BT deaths (Table 1), the time to death in the BT group increased from 13 hours (0.8 to 97 hours) to 36 hours (4.3 hours to 7 days) and the significant difference between the BT and the LEBT group was lost. After DOA exclusion, the early peak in trauma deaths (<1 hour) shifted to 1–3 days after injury (Figure 3).

## Discussion

Our findings indicate that clinical review is essential to confirm a trauma-related death, to define patients DOA and to determine the cause of death.

During the 5-year study period, 10.5% of the trauma deaths within 30 days were not related to the injury. This proportion is higher than in previous studies, which reported that 5% of in-hospital deaths [7] and 4/323 of 30-day mortalities [5] were not related to the preceding trauma. The fact that the majority of deaths not related to the injury were caused by an acute medical condition such as stroke and myocardial infarction does not imply that the patients were incorrectly included in the trauma registry. These patients were admitted with trauma team activation and met the criteria of trauma register inclusion. Therefore it is necessary to confirm a trauma related death in 30-day mortalities extracted from the trauma registry. However, the deaths not related to trauma in the current study were associated with minor injuries: therefore inclusion of these deaths did not affect mortality among ISS > 15.

Clinical review defined 17.6% of the patients who died from traumatic causes as DOA. The DOA criteria [8] were fulfilled in 90% of the patients suggesting that these criteria can be used to standardize the trauma registry inclusion of patients DOA. The percentage of patients DOA in the current study was lower than that reported by a large North American trauma center, where up to 32% of the patients were DOA or in traumatic arrest [7], but similar to that in a recent study from a trauma center in London (21.5%) [14]. The exclusion of patients DOA reduced overall mortality from 4.2% to 3.4% and among ISS >15, mortality changed from 17.5% to 14.8%. Furthermore, exclusion of patients DOA significantly increased the median age (64 to 71 years) and the time to death (24 to 43 hours), and decreased the median ISS (29 to 27) in the current study. Therefore, our results support the necessity of standardized DOA definitions allow for purposeful quality evaluations of deaths [8,15].

Traumatic brain injury was the most common cause of death, accounting for 58.6% of mortality excluding patients DOA. Comparable studies document similar high rates of CNS injuries as the main cause of death in Norway (67%) [16] but lower (47%) in Great Britain [14]. TBI as the leading cause of trauma death reflects a large proportion of blunt trauma. In the current study, trauma deaths in the patients who reached the hospital alive were due to blunt injury in 96% and the contribution of deaths due to penetrating trauma was negligible. The percentage of trauma deaths due to hemorrhage (16.3%) was in the same range or slightly lower as previous studies in Scandinavia [16,17]. The number of deaths in multiple organ failure 6.3% (16/253) was low in the current study. The reduction in the number of trauma deaths from multiple organ failure has been suggested to be due to improvements in critical care [18,19] as well as advances in resuscitation and operative treatment strategies [20].

The large proportion of deaths after LEBT among elderly trauma patients with pre-existing disease most likely contributed to the relatively high mortality rate, the high median age (71 years) and time to death (43 hours) in the current study. In the LEBT subgroup, the ASA class was higher compared to the other blunt trauma deaths and the most common cause of death was TBI and organ dysfunction. Our findings are in line with the recent observations by Kahl et al. who showed that increases in the number of elderly trauma patients with comorbid conditions have altered the characteristics (older age and lower ISS) and distribution of causes of deaths after trauma [20].

It can be argued that trauma deaths in elderly patients with comorbid conditions are largely unpreventable and that these patients are not in need of the resources of a trauma center [21]. A recent population-based study by Staudenmeyer et al., observed that most of the elderly (>55 years) patients admitted to a trauma center who died after trauma sustained minor injuries from low-energy mechanisms, but that treatment at trauma centers did not benefit these elderly patients as it did young patients [22]. However, in the present study, the patients whose injuries were caused by low-energy blunt trauma had a high median ISS (25) indicating that these patients were correctly triaged to the trauma center and should benefit from specialized care. Taken together, our result demonstrates that the elderly patient sustaining low-energy blunt trauma must be evaluated separately from other blunt trauma patients in order to allow for purposeful interpretations of blunt trauma deaths. Furthermore, each trauma center must find ways to identify this patient cohort to address the management of both the severely and nonseverely injured elderly trauma patient [20,22,23].

There are some strengths and limitations to the study. Retrospective design may potentially reduce the data quality. However, the data coverage in the CDR, trauma registry and clinical records is good with little information missing. The clinical review process is subjective by nature but the decisions were reached by consensus. Another limitation is that the effect of a new or pre-existing decision not to resuscitate is uncontrolled, especially in the case of severe brain injuries and/or significant co-morbidity in the elderly patient. This uncontrolled variable could have increased mortality in the current study.

## Conclusions

Clinical review of all trauma deaths was essential to interpret mortality. Thirty-day trauma mortality included 10.5% deaths not directly related to the injury and the exclusion of patients DOA significantly affected the unadjusted mortality rate, ISS, median age and time to death.

## Abbreviations

ISS: Injury severity score; DOA: Dead on arrival; AIS: Abbreviated injury scale; ASA: American Society of Anesthesiologists; SOFA: Sequential-related organ failure assessment.

## Competing interests

All authors declare no conflicts of interest.

## Authors’ contribution

PG, and LS, designed this study. PG, LS, and AO conducted the literature search. PG, and LS collected the data, which all authors analyzed and interpreted. PG and LS wrote the article and prepared the figures and tables. All authors provided critical revision of the manuscript and LS edited the final paper. All authors read and approved the final manuscript.

## References

[B1] Dutton RP Stansbury LG Leone S Kramer E Hess JR Scalea TM Trauma mortality in mature trauma systems: are we doing better? An analysis of trauma mortality patterns, 1997–2008 J Trauma 2010 69 620 626 10.1097/TA.0b013e3181bbfe2a 20093983

[B2] Pfeifer R Tarkin IS Rocos B Pape HC Patterns of mortality and causes of death in polytrauma patients–has anything changed? Injury 2009 40 907 911 10.1016/j.injury.2009.05.006 19540488

[B3] American College of Surgeons Committee on Trauma Resources for the Optimal Care of the Injured Patient 2006 Chicago, Illinois: American College of Surgeons

[B4] Ringdal KG Coats TJ Lefering R Di Bartolomeo S Steen PA Roise O Handolin L Lossius HM The Utstein template for uniform reporting of data following major trauma: a joint revision by SCANTEM, TARN, DGU-TR and RITG Scand J Trauma Resus 2008 16 7 10.1186/1757-7241-16-7 PMC2568949 18957069

[B5] Skaga NO Eken T Jones JM Steen PA Different definitions of patient outcome: consequences for performance analysis in trauma Injury 2008 39 612 622 10.1016/j.injury.2007.11.426 18377909

[B6] Pasquale MD Rhodes M Cipolle MD Hanley T Wasser T Defining “dead on arrival”: impact on a level I trauma center J Trauma 1996 41 726 730 10.1097/00005373-199610000-00022 8858036

[B7] Van Haren RM Thorson CM Curia E Schulman CI Namias N Livingstone AS Proctor KG Impact of definitions on trauma center mortality rates and performance J Trauma Acute Care Surg 2012 73 1512 1516 10.1097/TA.0b013e318270d40f 23188244

[B8] Powell DW Moore EE Cothren CC Ciesla DJ Burch JM Moore JB Johnson JL Is emergency department resuscitative thoracotomy futile care for the critically injured patient requiring prehospital cardiopulmonary resuscitation? J Am Coll Surg 2004 199 211 215 10.1016/j.jamcollsurg.2004.04.004 15275875

[B9] Esposito TJ Sanddal T Sanddal N Whitney J Dead men tell no tales: analysis of the use of autopsy reports in trauma system performance improvement activities J Trauma Acute Care Surg 2012 73 587 590 discussion 590–581 10.1097/TA.0b013e318265ce05 22929488

[B10] Romano PS McLoughlin E Unspecified injuries on death certificates: a source of bias in injury research Am J Epidemiol 1992 136 863 872 10.1093/aje/136.7.863 1442752

[B11] Brattstrom O Larsson E Granath F Riddez L Bell M Oldner A Time dependent influence of host factors on outcome after trauma Eur J Epidemiol 2012 27 233 241 10.1007/s10654-012-9651-4 22278437

[B12] Vincent JL Moreno R Takala J Willatts S De Mendonca A Bruining H Reinhart CK Suter PM Thijs LG The SOFA (sepsis-related organ failure assessment) score to describe organ dysfunction/failure. On behalf of the working group on sepsis-related problems of the European society of intensive care medicine Intens Care Med 1996 22 707 710 10.1007/BF01709751 8844239

[B13] Demetriades D Murray J Charalambides K Alo K Velmahos G Rhee P Chan L Trauma fatalities: time and location of hospital deaths J Am Coll Surg 2004 198 20 26 10.1016/j.jamcollsurg.2003.09.003 14698307

[B14] Chalkley D Cheung G Walsh M Tai N Deaths from trauma in London–a single centre experience Emerg Med J 2011 28 305 309 10.1136/emj.2009.085613 20581382

[B15] Newgard CD Fildes JJ Wu L Hemmila MR Burd RS Neal M Mann NC Shafi S Clark DE Goble S Nathens AB Methodology and analytic rationale for the American College of Surgeons Trauma Quality Improvement Program J Am Coll Surg 2013 216 147 157 10.1016/j.jamcollsurg.2012.08.017 23062519

[B16] Soreide K Kruger AJ Vardal AL Ellingsen CL Soreide E Lossius HM Epidemiology and contemporary patterns of trauma deaths: changing place, similar pace, older face World J Surg 2007 31 2092 2103 10.1007/s00268-007-9226-9 17899256

[B17] Groven S Eken T Skaga NO Roise O Naess PA Gaarder C Long-lasting performance improvement after formalization of a dedicated trauma service J Trauma 2011 70 569 574 10.1097/TA.0b013e31820d1a9b 21610344

[B18] Acosta JA Yang JC Winchell RJ Simons RK Fortlage DA Hollingsworth-Fridlund P Hoyt DB Lethal injuries and time to death in a level I trauma center J Am Coll Surg 1998 186 528 533 10.1016/S1072-7515(98)00082-9 9583692

[B19] Sauaia A Moore FA Moore EE Moser KS Brennan R Read RA Pons PT Epidemiology of trauma deaths: a reassessment J Trauma 1995 38 185 193 10.1097/00005373-199502000-00006 7869433

[B20] Kahl JE Calvo RY Sise MJ Sise CB Thorndike JF Shackford SR The changing nature of death on the trauma service J Trauma Acute Care Surg 2013 75 195 201 10.1097/TA.0b013e3182997865 23823614

[B21] Cayten CG Stahl WM Agarwal N Murphy JG Analyses of preventable deaths by mechanism of injury among 13,500 trauma admissions Ann Surg 1991 214 510 520 discussion 520–511 10.1097/00000658-199110000-00015 1953102 PMC1358558

[B22] Staudenmayer KL Hsia RY Mann NC Spain DA Newgard CD Triage of elderly trauma patients: a population-based perspective J Am Coll Surg 2013 217 569 576 10.1016/j.jamcollsurg.2013.06.017 24054408 PMC3839622

[B23] Grossman MD Miller D Scaff DW Arcona S When is an elder old? Effect of preexisting conditions on mortality in geriatric trauma J Trauma 2002 52 242 246 10.1097/00005373-200202000-00007 11834982

